# On-Demand ICS + FABA Combinations in 6–11-Year-Old Children

**DOI:** 10.3390/jcm12237270

**Published:** 2023-11-23

**Authors:** Elsy M. Navarrete-Rodríguez, Blanca E. Del-Rio-Navarro, Désirée Larenas-Linnemann, Jose A. Castro-Rodriguez

**Affiliations:** 1 Secretaría de Salud, Hospital Infantil de México Federico Gómez, Servicio de Alergia e Inmunología Clínica, Ciudad de Mexico 06760, Mexico; draenavarrete@gmail.com (E.M.N.-R.); blancadelrionavarro@gmail.com (B.E.D.-R.-N.); 2 Centro de Excelencia en Asma y Alergia Larenas, Hospital Médica Sur, Ciudad de Mexico 14050, Mexico; marlar1@prodigy.net.mx; 3 Departamento de Neumología Pediátrica, División de Pediatría, Facultad de Medicina, Pontificia Universidad Católica de Chile, Santiago 8331150, Chile

**Keywords:** fast-acting beta-agonist, asthma treatment, anti-inflammatory rescue therapy, school children

## Abstract

In recent years, some new concepts have been added to asthma treatment such as “anti-inflammatory reliever” (β2-agonist use associated to an inhaled corticosteroid (ICS) as a reliever treatment) that combines the benefits of both therapies and provides short- and long-term benefits for treatment in asthma patients. Robust evidence has been presented in patients over 12 years, and the main changes in the international guidelines for asthma treatment were originally made in this age group. However, a few suggestions have been added to treatments in younger patients, in part because of the scarce evidence that exists in this group. We aim to analyze the information regarding the utilization of ICS + fast-acting beta-agonist (FABA) combination in children between 6 and 11 years. Although up until today only three published trials exist (two studies use beclomethasone + albuterol and one study uses budesonide + formoterol), they provide significant information on the benefits of ICS + FABA use in this population.

## 1. Introduction

Asthma is a frequent, chronic, heterogeneous disease of the respiratory tract, usually characterized by chronic airway inflammation, which causes a limitation in the expiratory flow that results in symptoms that vary in intensity like wheezing, shortness of breath, chest tightness, and cough [1,2].

The concept of disease variability related to airway inflammation allowed, for many years, treatment to be divided into two groups of medications: controllers and relievers, which, added to the utilization of treatment steps, enabled the personalization of the therapeutic options according to severity of the pathology to reduce symptoms, prevent exacerbations, improve lung function, and reduce mortality. But, as the severity of asthma symptoms and the level of airway inflammation vary over time, in recent years, this way of dividing treatment has changed, and new concepts have been added such as “anti-inflammatory reliever” (reliever β2-agonist use associated to an ICS as an on-demand controller treatment) [1] that combines the advantages of both therapies and provides short- and long-term benefits for treatment in asthma patients.

To understand the benefit of adding to the ICS a reliever drug like a fast-acting beta-agonist (FABA) such as salbutamol or formoterol, as well as to understand the new indication in the use of medications in this disorder, we must look back at the history of asthma treatment. At the beginning of the 1900s, asthma treatment was a big challenge, especially because there were no portable devices that allowed delivering medication at pulmonary level in an adequate form. The first metered-dose inhaler (MDI) portable devices available for inhalation in the treatment of asthma contained epinephrine and isoprenaline that were effective and quick to reach bronchodilatation [3]. Misfortunately, along with the rise in sales of these devices, an increase in the mortality rate of the disease was observed [4]. Consequently, asthma in the 1980s was considered as a disease with a high mortality rate, associated with the presence of severe adverse treatment effects, making it clear that asthma is an inflammatory disorder of the airways and the wide acceptability of the bronchodilator reliever β2-agonist medication as a monotherapy resulted in the delay of the start of anti-inflammatory treatment, which invariably led to an increased risk of severe exacerbations and death. Finally, it became clear that asthma was an inflammatory disorder of the airways and hence the importance of inhaled corticosteroids as mainstay maintenance therapy was highlighted.

Consequently, the asthma mortality rate has decreased in the last decades and well-defined risk factors for severe exacerbations are now known such as previous near-fatal exacerbations, repetitive emergency visits or hospitalizations for asthma in the last year, three or more different medication classes needed to maintain asthma control, psychiatric diseases, use or abuse of drugs and/or alcohol, as well as intensive and excessive use of β2-agonists and the lack of adherence to the maintenance treatment or follow up [5,6]. However, patients with very occasional symptoms are still prone to use only reliever drugs such as short-acting β2-agonist (SABA), as these lead to a false sensation of disease control, ignoring that their symptoms are only the tip of the iceberg of changes that are produced in the lower airways by the inflammation. Moreover, over three decades (1996–2015), no overall change in the adherence rates has been observed, and, for pediatric asthma, the adherence in studies using objective measures is approximately 45% [7]. Thus, this is the group of patients in which special attention is needed because, despite their seemingly mild and sporadic symptoms, they are the ones at risk of having severe exacerbations [1].

Even though the harmful effects of the use of β2-agonists as monotherapy for asthma control have been known for many years, changes in the international guidelines were minimal for a long time, especially regarding treatment in patients with sporadic symptoms (STEP 1) [8], as this is a subgroup not easy to define. Also, as their exacerbation rate is low, a large number of patients is needed for trials to be adequately powered. It was not until 2019 that an important amendment was made in the Global Initiative for Asthma (GINA) and the recommendation was to add an ICS to β2-agonist for the treatment of acute symptoms, already from STEP 1 onward. With such a treatment schedule, a decrease in the risk of exacerbations and in the use of oral corticosteroids (OCS) has been demonstrated in adolescent and adults [8,9]. As there was very little evidence for such therapy in children, the changes were only recommended in adolescents–adults.

However, it is of particular interest to know what exactly happens in school children as asthma therapy primarily focusses on reducing the frequency of exacerbations, because pediatric asthma is generally characterized by a high rate of exacerbations. Several studies demonstrated the relationship between exacerbation frequency and lung function decline in milder cases of adults, [10] children, and adolescents with asthma [11]. However, the main lung function declination occurred mostly in children rather than in adolescents or adults [11]. Recently, the Severe Asthma Research Program (SARP) demonstrated the same relationship in boys, but not in girls [12].

**Current recommendations (Table 1):**

### 1.1. Step 1 Treatment: Use an ICS Every Time You Use a Short-Acting β2-Agonist SABA

Regarding the use of an ICS plus a SABA as reliever therapy for patients under the age of 12, the recommendation is mainly based on the TREXA study by Martinez et al. published in 2011 [13]. It is a double-blind trial, with four treatment arms and a two-by-two factorial design in children and adolescents between 6 and 18 years with a history of mild persistent asthma during the previous 2 years. Patients were randomly assigned to one of the four treatment groups: Group 1: twice-daily beclomethasone BDP with BDP plus albuterol as rescue (combined group); Group 2: twice-daily BDP with placebo plus albuterol as rescue (daily BDP group); Group 3: twice-daily placebo with BDP plus albuterol as rescue (rescue BDP group); and Group 4: twice-daily placebo with placebo plus albuterol as rescue (placebo group). Only for clarification, in the study, the BDP and albuterol were administered in separate devices.

**Figure 1:**
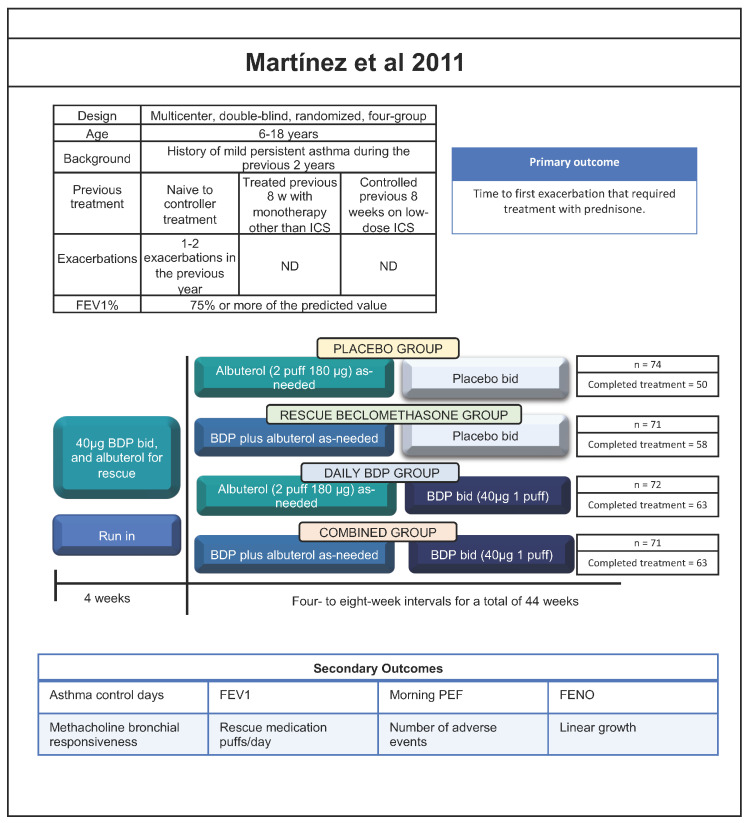

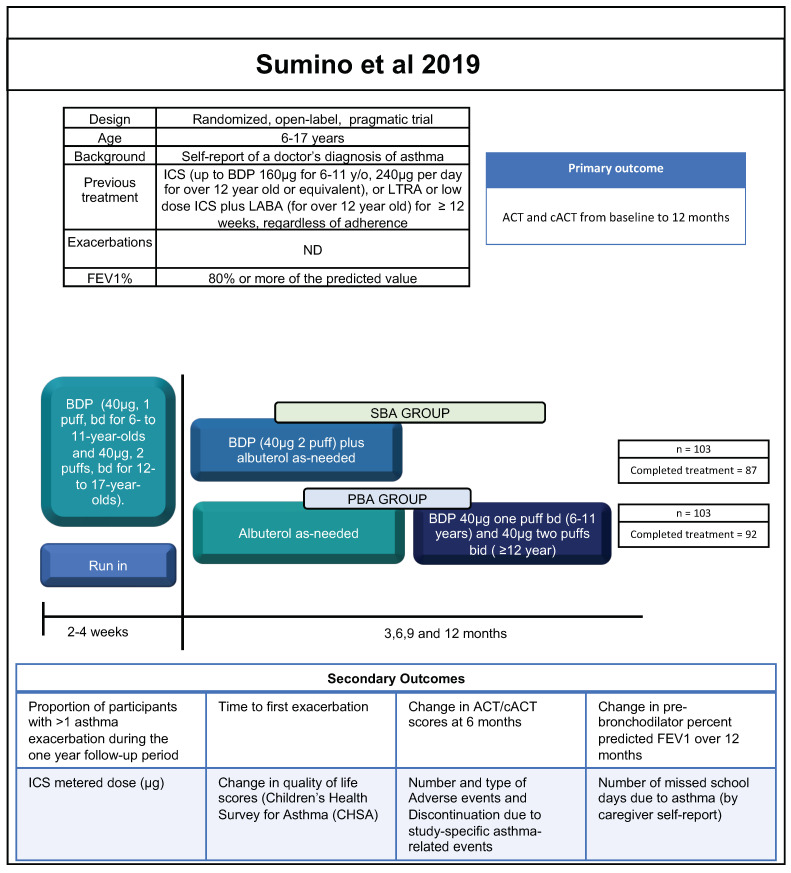

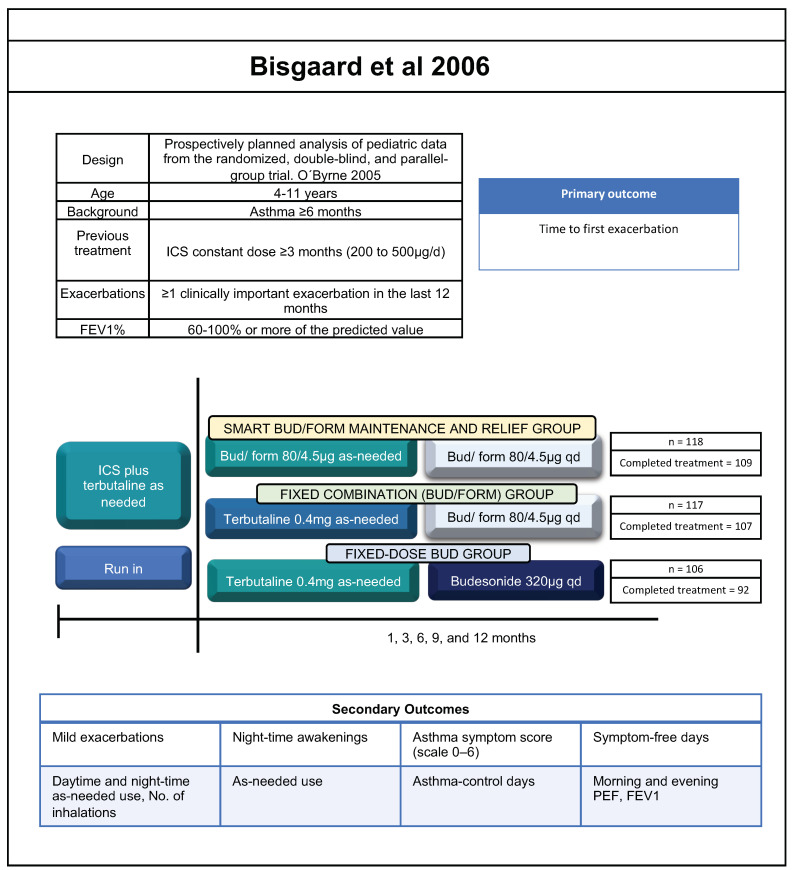
Summary of studies for the use of ICS + bronchodilator in children under 18 years of age [13,14,15,16]. Abbreviations: ND, not done; FEV_1_, forced expiratory volume in first second; ICS, inhaled corticosteroid; PEF, peak expiratory flow; SABA, short-acting β2-agonist; FENO, fractional exhaled nitric oxide; LABA, long-acting β2-agonist; LTRA, leukotriene receptor antagonist; BDP, beclomethasone dipropionate; BID, twice daily; QD, once daily.

The primary efficacy outcome was the time to the first exacerbation that required treatment with prednisone (Figure 1 and Table 2 and Table 3). This study allowed us to identify benefits in the use of a combined therapy of ICS and SABA as reliever, observing the following:

**In favor of the use of combined reliever therapy:**

- Frequency of treatment failures (the requirement for a second dose of prednisone within any 6-month period) was higher in the placebo group vs. the twice-daily BDP with BDP plus albuterol as rescue (combined group): 23% (95% CI [14–34], n = 17) vs. 8.5% ([2–15], n = 6), *p* = 0.024.
- Compared with the placebo group, the hazard ratio for asthma exacerbations was lower in the rescue BDP group (0.62 [0.37–1.05], *p* = 0.073).

**Against the use of combined reliever therapy:**

- No difference in asthma control days, morning peak expiratory flow (PEF), fractional exhaled nitric oxide (FENO), methacholine bronchial responsiveness, or quality of life in patients using for the relief of their symptoms albuterol vs. the use of SABA + ICS (rescue beclomethasone group).
- There was a significant decrease regarding the prebronchodilator percentage predicted FEV_1_ in the placebo and rescue BDP group, although the decrease was higher in the group using only albuterol (−6.6%, SD 1.7, *p* = 0.0001) vs. (−4.1%, SD 1.8, *p* = 0.024).

### 1.2. Step 2 Treatment: Continuous ICS vs. ICS + a Fast-Acting Beta-Agonist (FABA) as Needed

Patients with infrequent symptoms are often poorly adherent to controller drugs, especially because usually they do not consider this stage of asthma as a pathology that requires a continuous treatment. This exposes them to the risk of SABA overuse that only provides a bronchodilator effect without an anti-inflammatory effect. It has been observed in double-blind placebo-controlled studies that in adolescents and adults, the use of as-needed combined therapy (ICS + formoterol) compared with maintenance daily ICS and SABA rescue led to an equal risk of exacerbations [17,18], and in open and pragmatic studies this approach was even better than the continuous daily ICS treatment [19] (Beasley et al., 2019) [20].

Two studies compared daily maintenance ICS vs. as-needed ICS + FABA in children under 12 years old, the TREXA trial [13], and the study published by Sumino et in 2019 [14]. As was explained before, the TREXA study fixed-combination group [13] included a regular treatment group (ICS twice a day) and rescue BDP group (ICS + albuterol only as reliever without regular treatment). Sumino’s study was a randomized, open-label, two-arm, pragmatic trial in African American children, 6 to 17 years old, with mild asthma (prescribed low-dose ICS, leukotriene receptor antagonist, or low-dose ICS plus LABA (for 12 to 17 year olds). In that study [14], the participants were divided into two groups: Group 1: symptom-based adjustment (SBA): the participants were instructed to take two puffs of beclomethasone 40 μg (total 80 μg) each time they took albuterol when they experienced symptoms; and Group 2: provider-based guideline-directed adjustment (PBA): participants were instructed to take one puff of beclomethasone 40 μg twice daily (for 6 to 11 year olds) or two puffs of beclomethasone 40 μg twice daily (for 12 to 17 year olds). The primary efficacy outcome was the change in Asthma Control Test (ACT) score (ACT for 12 to 17 year olds; childhood ACT [cACT] for 6 to 11 year olds) from baseline to 12 months (Figure 1 and Table 2 and Table 3).

When comparing the groups of continuous daily ICS vs. ICS + FABA as needed, the following results from these two trials [13,14] were:

**In favor of the use of continuous ICS therapy:**

- Individuals in the daily beclomethasone groups had a lower FeNO during the trial (*p* < 0.0001) [13].
- FEV_1_ decreased in daily ICS and ICS + FABA groups, although this was only significant in the second one (−4.1%, 1.8, *p* = 0.024) [13].

**Against the use of continuous ICS therapy:**

- The probability of a first exacerbation by the end of the trial was reduced by 28% [18–40] (n = 20) in the daily BDP group and 35% [24–47] (n = 25) in the rescue BDP group, both compared with the placebo group, although the differences were not significant, and no comparison was made between those two groups [13].
- No difference between continuous ICS therapy vs. ICS + FABA as needed was observed in terms of: asthma control days, morning PEF, methacholine bronchial responsiveness, quality of life, and treatment failures [13].
- There was no significant difference between SBA vs. PBA groups in the exacerbations, time to the first exacerbation, asthma control questionnaire (ACQ-5) score, self-reported, missed school days per year, FEV_1,_ and quality of life [14].
- The use of BDP (μg/month) was greater in the PBA group vs. SBA group 1961 μg/month (1681–2241 μg) vs. 526 μg/month (413–639 μg), respectively, *p* < 0.0001 [14].
- Children in the daily BDP group grew 1·1 cm (SD 0.3) less than the children in the placebo group (*p* < 0.0001), while the difference between rescue BDP and placebo was not significant [13].

### 1.3. Step 3 Treatment: Use of MART Therapy

The use of maintenance and reliever therapy (MART) is currently recommended by GINA 2023 [1] for children older than 6 years, although the Coordinating Committee of the National Asthma Education and Prevention Program (NAEPPCC) recommend its use from 4 years onward [21]. These recommendations are based on the article published by Bisgaard et al. in 2006 [15], which is a prospectively planned post-hoc analysis of pediatric data from the pediatric protocol in a 12-month, randomized, double-blind, and parallel-group trial published by O’ Byrne in 2005 [16]. Children aged 4 to 11 years with asthma treated with ICS (any brand) at a constant dose for ≥3 months (200 to 500 μg/d) and at least one clinically important asthma exacerbation in the 12 months before study entry were enrolled. Patients were randomly assigned to one of the three treatment groups: Group 1: once-daily budesonide–formoterol (BUD/FORM) (Symbicort^®^ via Turbuhaler^®^) 80/4.5 μg plus additional doses as needed (MART group); Group 2: once-daily BUD/FORM 80/4.5 μg plus terbutaline 0.4 mg for rescue medication (fixed-combination group); and Group 3: once-daily budesonide (BUD) 320 μg plus terbutaline 0.4 mg for rescue medication (fixed-dose budesonide group) [15]. The primary outcome measure was the time to the first exacerbation (Figure 1 and Table 2 and Table 3).

**In favor of the use of the MART strategy:**

- The risk of experiencing a severe asthma exacerbation was 66% lower when the MART approach was used versus the fixed-combination group (hazard ratio (HR): 0.34, [95% CI 0.19–0.60]), and 51% lower than the fixed-dose budesonide group (HR: 0.49 [0.27–0.90]). Also, there were less exacerbations requiring medical intervention in the MART group 8% vs. 20% (fixed-dose budesonide group) and 31% (fixed-combination group). Morning and evening PEF were significantly better in the MART group vs. the fixed-dose budesonide group, but there was no difference between the utilization of MART vs. the fixed-combination group.

## 2. Discussion

With the advent of new trials, the benefits of the use of ICS + FABA combinations have become more evident, which has made it possible to position this type of therapy among those preferred in patients over 12 years of age. While studies in children under 12 have been rare, in this review, we described the only three trials referred on the strategies or guidelines for the treatment of asthma in this age group: two studies used albuterol + beclomethasone, and one used formoterol + budesonide.

The benefits of using an anti-inflammatory rescue have been overwhelming in adolescents and adults; however, in children between 6 and 11 years, the only benefits identified with this approach are a decrease in the HR for asthma exacerbations and a decrease in the frequency of asthma treatment failure when compared with salbutamol alone. It is possible that the results of this kind of therapy in school children (6–11 years) are not so different from those in adolescents and adults; nevertheless, we do not know how valid it is to translate the results of this intervention to another age group, and the main problems would perhaps lie in accounting for the extra doses of ICS and identifying if this increase in the use of ICS could have long-term consequences, especially concerning final height and if the minor clinical changes, especially demonstrated on pulmonary function, are important not only for the present but for future lung health.

When discussing the first steps of asthma treatment, a crucial point is the need to establish clear limits for step up. For this approach, we must clarify when to tell the patient being rescued with ICS + FABA that they are not controlling their asthma and must move on to Step 2 or Step 3 and use continuous ICS or ICS + formoterol. GINA gave some recommendations about the initial treatment; however, in general, GINA provides little information on when to change the therapy in these new approaches. For example, a patient who uses anti-inflammatory rescue and who is losing control could be rescued 3 to 4 times a week before step up. Taking into consideration the issue of low adherence to daily ICS (around 45%) described in these patients [7], their treatment probably falls in poorly adherent to therapy in Step 3. Nevertheless, if we allow this to happen without supervision, the message that we are giving to our patient could be unclear.

With the advent of new combined therapies, it should be considered that we are now talking about ICS + FABA. However, we can use this combination in various presentations: ICS + SABA in separate devices, ICS+ SABA in one device, and ICS+ formoterol in one device. In the case of ICS + SABA in separate devices, it is essential to clearly explain to the patient how many puffs of ICS should be taken for each puff of salbutamol. We also consider that not all countries have all the ICS dosages, mainly to keep the doses low. In addition, we need to answer other questions, such as whether the administration of medications together or separately is the same, or if ICS + formoterol is better than ICS + salbutamol specifically for this age group. Another critical point is to establish recommendations for the use of ICS in the case of the use of nebulized therapy. Many questions may remain unsolved yet, the most important being when to decide that this approach is not working. Remember that the doses used in anti-inflammatory rescue are low, and if the patient is not controlled with low ICS doses, it will be important to move them to higher ICS doses.

It is also essential to consider that not all ICS are the same and that there are substantial differences in their action, bioavailability, and growth influence. Fluticasone is least implicated in height alteration in young children [22], although these three available studies were carried out with beclomethasone and budesonide.

Regarding Step 2, there is information that supports both the use of continuous ICS therapy and the use of intermittent treatment with an ICS + FABA combination in patients over 12. This recommendation differs for children between 6 and 11 years, and it is preferred to maintain the ICS continuously as the primary therapy. Further studies exploring the use of intermittent therapy will be attractive, especially in poorly adherent patients; although, once again, we insist on establishing limits for its use vs. switching to Step 3 of treatment.

In Step 3, MART therapy uses continuous, very low doses of ICS + formoterol (80 μg budesonide delivered daily), adding extra amounts in case of symptoms. In day-to-day practice, many physicians use this concept of MART as a synonym for using the same maintenance and rescue device, although this is not exactly true, and it is essential to know these details. The only study that led to the inclusion of MART therapy in children under 12 years of age is the Bisgaard study [15]. In this study, 80 μg of budesonide was used once a day, that is, a very low dose; the question arises if this strategy is adequate or if these patients initially needed higher steroids. Also, if the results are similar, using the same device with formoterol as FABA (MART therapy) or ICS + albuterol can be used in a patient-activated, reliever-triggered inhaled glucocorticoid therapy (PARTICS therapy) [23]. Therefore, it will be essential to decide the best long-term treatment objective that allows integrating objective values such as lung function and long-term exacerbations and subjective results of quality of life and well-being in both patients and their caregivers. More information remains to be obtained, and even though the information is oriented towards the benefits of these combinations, we must always encourage new research that allows us to consolidate the evidence of these benefits not only to patients older than 12 years but also to younger schoolchildren (6–11 years).

The strengths of this review are to summarize all the information about all studies published on the use of ICS + FABA in children under 12 years of age, visualize that the international recommendations or guidelines have been made based on very few trials (only three), and reinforce the necessity of acting cautiously in this age group and not generalizing the recommendations. The main limitation is that other treatment options, such as the addition of montelukast or the increase in ICS to medium doses, or the use of the combination of ICS + LABA or adding tiotropium, as viable options is outside the objectives of this review.

## 3. Conclusions

The combinations of ICS + FABA have been demonstrated to be beneficial in patients with asthma over 12 years of age in Steps 1, 2, 3. In children between 6 and 11 years, there is little, but still significant, information on the benefits of their use. More research is required in this age group to know the short- and long-term benefits and harms of using these combinations.

## Figures and Tables

**Table 1:** Treatment recommended by the main reference guidelines.

	GINA 6–11 Years [1]			GEMA ≥ 4 Years [2]			BTS/SIGN (Pediatric Treatment) [5]		
	Step 1	Step 2	Step 3	Step 1	Step 2	Step 3	Step 1	Step 2	Step 3
**Preferred Controller**	Low-dose ICS taken whenever SABA taken	-Daily low-dose ICS	-Low-dose ICS-LABA. -Medium-dose ICS -Very-low-dose ICS + Formoterol maintenance and reliever (MART)	No treatment needed	Daily low-dose ICS	-Medium-dose ICS -Low-dose ICS-LABA	No treatment needed	Very-low- (pediatric) dose ICS	-Very-low- (pediatric) dose ICS + LABA or LTRA (children ≥ 5) -Very-low- (pediatric) dose ICS + LTRA (children < 5)
**Other controller options**	Consider daily low-dose ICS	-Daily leukotriene receptor antagonist. -Low-dose ICS taken whenever SABA taken	Low-dose ICS + LTRA		Daily LTRA	Low-dose ICS + LTRA			
**Reliever**	As-needed SABA	As-needed SABA	-As-needed SABA -Low-dose ICS–formoterol reliever for MART	As-needed SABA	As-needed SABA	As-needed SABA	As-needed SABA	As-needed SABA	As-needed SABA

ICS = Inhaled corticosteroid, SABA = Short-acting β2-agonist, LTRA = Leukotriene receptor antagonist.

**Table 2:** Methodology of studies steps 1 and 2 in patients under 12 years old.

				TREXA 2011 [13]			SUMINO 2019 [14]		BISGAARD 2006 [15]	
**Methods**	**Design**			Multicenter, double-blind, randomized, four-group			Randomized, open-label, pragmatic trial		Prospectively planned analysis of pediatric data from randomized, double-blind, and parallel-group trial. O’Byrne 2005 [16]	
	**Participants**	**Age**		6–18 years			6–17 years		4–11 years	
		**Asthma diagnosis**		History of mild persistent asthma during the previous 2 years			Self-report of a doctor’s diagnosis of asthma		Asthma history ≥ 6 months	
		**Treatment step**		2			2 (6–11 years), 2–3 (2–17 years)		3	
		**Main Inclusion Criteria**	**Previous treatment**	Naive to controller treatment	Treated previous 8 w with monotherapy other than ICS	Controlled 8 weeks on low ICS	ICS (up to BDP 160 mcg for 6–11 y/o, 240 mcg per day for over 12 year old or equivalent), or LTRA or low dose ICS plus LABA (for over 12 year old) for ≥12 weeks, regardless of adherence		ICS (any brand) at a constant dose for ≥3 months (200 to 500 mcg/d)	
			**Symptoms**	ND			Asthma Evaluation Questionnaire Score (AEQ) 1 up to score 2 in 2 out of 3 questions with total score ≤ 5.		Eight or more inhalations of terbutaline in the last 10 days of run-in and up to seven inhalations on any 1 day	
			**Exacerbations**	1–2 previous year	ND	ND	ND		One clinically important in the last 12 months.	
			**FEV1%**	75% or more of the predicted value			≥80% predicted		60 to 100% predicted	
	**Run in**			4w run-in period, 40mcg BDP bd, and albuterol for rescue. Placebo rescue inhaler to be used along with albuterol for symptoms.			2- to 4w. BDP (40 mg, 1 puff, bd for 6–11 year and 40 mg, 2 puffs, bd for 12- to 17-year-olds). Telephone-based education (2–4 sessions).		Previous ICS plus terbutaline as needed	
	**Additional treatment permitted if worsening**			Albuterol for the prevention of exercise-induced asthma			In PBA group.- Subsequent dosing adjustments were made by the physician according national guidelines		Not more than 8 inhalations of the medication 1 day. If the patient needed more medication, the investigator had to be contacted.	
	**Adherence to treatment**			Electronic measurements review at each visit.			ND		ND	
	**Trial Visits**			Four- to eight-week intervals for a total of 44 weeks			6: 0, randomization, 3,6,9 and 12 months.		1, 3, 6, 9, and 12 months	
	**Masking**			Yes			One unmasked study staff assigned to each provider office for randomization, procedures and deal with assignment related tasks.		Yes	
	**Treatment duration**			44 weeks			12 Months		12 months	
	**Groups**			1. Placebo bd with placebo plus albuterol (2 puff 180 μg) as-needed. **PLACEBO GROUP** (ALB)	n = 74					
					Received treatment = 74					
					Completed treatment = 50					
				2. Placebo bd with BDP plus albuterol as-needed. **RESCUE BDP GROUP**	n = 71		1. BDP 40 mcg two puffs each time they needed to take albuterol. Symptom-based adjustment. **SBA GROUP**.	n = 103		
					Received treatment = 71			Received treatment 103		
					Completed treatment = 58			Completed treatment = 87. Analyzed: 103		
				3. BDP bd (40 mcgs 1 puff) with placebo plus albuterol as-needed (2 puff 180 μg). **DAILY BDP GROUP**	n = 72		2. BDP 40 mcg one puff bd (6–11 years) and 40 mcg two puffs bd (≥12 year) Provider-based guideline-directed adjustment **PBA GROUP.**	n = 103		
					Received treatment = 72			Received treatment 103		
					Completed treatment = 63			Completed treatment = 92. Analyzed: 103		
				4. BDP bd (40 mcgs 1 puff), with BDP (2 puff 40 mcgs each) plus albuterol (2 puff 180 μg) as rescue. **COMBINED GROUP**	n = 71					
					Received treatment = 71					
					Completed treatment = 63					
									BUD/FORM Turbuhaler 80/4.5 μg qd plus additional doses as needed (SMART)	n = 106
										Received treatment = 106
										Completed treatmente = 92 Anallyzed = 106
									BUD/FORM 80/4.5 μg qd plus terbutaline 0.4 mg for rescue (FIXED-COMBINATION GROUP)	n = 117
										Received treatment = 117
										Completed treatmente = 107 Anallyzed = 117
									Fourfold-higher maintenance dose of BUD 320 μg qd plus terbutaline 0.4 mg for rescue (FIXED-DOSE BUDESONIDE GROUP).	n = 118
										Received treatment = 118
										Completed treatmente = 109 Anallyzed = 18
	**Primary outcome variable**			Time to first exacerbation that required treatment with prednisone.			ACT and cACT from baseline to 12 months.		Time to first exacerbation	

w = weeks, y = years, ICS = inhaled corticosteroid, BDP = beclomethasone dipropionate, LTRA = leukotriene receptor antagonist, LABA = long acting β2-agonist, BUD = budesonide, FORM = formoterol, bd = twice a day, PBA group = provided-based guideline direct adjustement group, SBA GROUP = Symptom-based adjustment, ND = not done, ALB = albuterol, COMB = Combination, ACT = asthma control test.

**Table 3:** Main results of studies steps 1 and 2 in patients under 12 years old.

		SABA Alone vs. ICS + SABA	Regular ICS Treatment vs. ICS + SABA as Needed		SMART vs. Regular ICS Treatment + Terbutaline vs. Forudold-Higer ICS Treatment
		TREXA 2011 [13]	TREXA 2011 [13]	SUMINO 2019 [14]	BISGAARD 2006 [15]
**Symptoms**	**Exacerbations**	RESCUE BDP GROUP better than PLACEBO GROUP HR (0.62 0.37–1.05, *p* = 0.073).	ND	No significant difference SBA GROUP vs. PBA GROUP *p* = 0.62	SMART better than FIXED-COMBINATION GROUP (*p* < 0.001) and FIXED-DOSE BUDESONIDE (*p* = 0.022) (0.41, 0.76, 0.48)
	**Time to the first exacerbation**	No difference between RESCUE BDP GROUP vs. PLACEBO GROUP HR 0.62 (0.37–1.05) *p* = 0.073	RESCUE BDP GROUP trend worse than DAILY BDP GROUP (NSR)	No significant difference SBA GROUP vs. PBA GROUP *p* = 0.49	SMART betther than FIXED-COMBINATION GROUP (*p* < 0.001) and FIXED-DOSE BUDESONIDE GROUP (*p* = 0.02)
	**ACQ-5 score**	ND	ND	No significant difference between SBA GROUP vs. PBA GROUP *p* = 0.10	ND
	**Asthma Control Days**	No difference reported between groups.	No difference between RESCUE BDP GROUP vs. DAILY BDP GROUP	ND	FIXED-DOSE BUDESONIDE GROUP trends to be better than SMART (*p* = 0.14) and FIXED-COMBINATION GROUP (*p* = 0.6) (50.8 vs. 57.0 vs. 60.6)
	**Self-reported, missed school days per year**	ND	ND	No significant difference between SBA GROUP vs. PBA GROUP *p* = 0.84	ND
**Pulmonary function test**	**FEV1**	RESCUE BDP GROUP trends better than PLACEBO GROUP (SNR)	RESCUE BDP GROUP and PLACEBO GROUP worse than DAILY BDP GROUP and COMBINED GROUP *p* = 0·024	No significant difference between SBA GROUP vs. PBA GROUP *p* = 0.14	FIXED-COMBINATION GROUP trends to be better than FIXED-DOSE BUDESONIDE GROUP (*p* = 0.43) and SMART (*p* = 0.094) (1.70 L vs. 1.76 L vs. 1.86 L)
	**Morning PEF**	No difference reported between groups.	No difference reported between groups.	ND	SMART better than FIXED-DOSE BUDESONIDE GROUP (*p* = 0.0019) and trends to be better than FIXED-COMBINATION GROUP (*p* = 0.22) (255 vs. 238 vs. 242 L/min)
	**FENO**	No difference between RESCUE BDP GROUP vs. PLACEBO GROUP (SNR)	RESCUE BDP GROUP and PLACEBO GROUP worse than DAILY BDP GROUP and COMBINED GROUP	ND	ND
	**Methacholine bronchial responsiveness**	No difference between groups.	No difference between groups.	ND	ND
**Rescue medication**	**Beta-agonist-containing actuations per day**	ND	RESCUE BDP GROUP trend worse than DAILY BDP GROUP (SNR)	ND	ND
**Glucocorticoid treatment**	**ICS metered dose (μg)**	ND	ND	SBA GROUP better than PBA GROUP *p* < 0.001	FIXED-COMBINATION GROUP trends to be better than SMART and FIXED-DOSE BUDESONIDE GROUP (80 mcg/dia vs. 126/7.1 mcg/d vs. 320 mcg/d) (SNR)
**Quality of life**	**AQLQ score**	No difference reported between groups.	No difference reported between groups.	No significant difference SBA GROUP. vs. PBA GROUP	ND
**Adverse events**	**Adverse events**	ND	ND	No significant difference SBA GROUP vs. PBA GROUP (SNR)	SMART trends to be better than FIXED-DOSE BUDESONIDE GROUP and FIXED-COMBINATION GROUP (2, 5, 16) (SNR)
	**Discontinuation due to study-specific asthma-related events**	ND	ND	No significant difference SBA GROUP vs. PBA GROUP	ND
	**Linear Growth**	No difference between RESCUE BDP GROUP vs. PLACEBO GROUP −0.3 cm (0.2) *p* = 0.26	RESCUE BDP GROUP trends better than DAILY BDP GROUP (SNR)	ND	SMART better than FIXED-DOSE BUDESONIDE GROUP *p* < 0.01 5.3 (1.0–14.0) vs. 4.3 (−2.0–15.0) FIXED-COMBINATION GROUP better than FIXED-DOSE BUDESONIDE GROUP *p* < 0.01 5.4 (−4.0–12.0) vs. 5.3 (1.0–14.0)
	**Treatment failures**	ND	No significance is reported between groups	ND	ND
**Adherence**	**Adherence to the twice-daily, blinded maintenance regimen**	ND	ND	ND	ND

HR = Hazard Ratio, ND = Not done, BDP = beclomethasone dipropionate, BUD = budesonide, ACQ-5 = Asthma control Questionaire, FEV1 = forced expiratory volume in 1st second, SNR = statistical significance not reported, PEF = peak expiratory flow, FENO = fractional exhaled nitric oxide, AQLQ = asthma-related quality of life questionnaire.
